# Transcriptomic and Proteomic Study on Animal Venom: Looking Forward

**DOI:** 10.3390/toxins18050213

**Published:** 2026-05-01

**Authors:** Paulo Lee Ho

**Affiliations:** Instituto Butantan/Fundação Butantan, São Paulo 05503-900, SP, Brazil; paulo.ho@fundacaobutantan.org.br

Transcriptomic and proteomic studies concerning venom and venom glands have provided major breakthroughs in the characterization and knowledge of global crude venom compositions and have also allowed for the identification of new toxins and activities. Venom gland transcriptomes (mRNA) and proteomes (proteins) share a high degree of concordance regarding the major toxins produced, meaning the most heavily transcribed genes in the gland generally correspond to the most abundant proteins in the venom. These approaches are based heavily on specific techniques that allow for in-depth characterization and knowledge of global crude venom compositions like DNA sequencing and mass spectrometry, respectively. However, the transcriptome–proteome relationship is not perfectly linear, with studies often showing that venom gland transcriptomes contain a larger, more diverse array of “potential” toxin transcripts, while the proteome reflects only the “actual” translated products identified [1,2,3,4,5,6,7,8,9].

Commonalities between the venom gland transcriptome and proteome include:

- Dominant Toxin Representation: In both profiles, venom composition is dominated by a few major toxin superfamilies, such as phospholipases (PLA2s), metalloproteinases (SVMPs), and three-finger toxins (3FTxs), depending on the species analyzed.
- High Expression and Production Levels: The mRNA levels (transcriptome) in the gland generally correlate with the protein abundance (proteome) in the raw venom, indicating that transcription directly dictates the high volume of toxin production. Major transcripts correlate with the major toxins expressed.
- Structural Similarities and Fold Types: Both approaches commonly identify the same characteristic cysteine-rich toxins, though both approaches do not provide direct tertiary or quaternary structures.
- Multifamily Organization: Both profiles reveal that toxins are organized into similar families and subfamilies, often with high levels of sequence similarity between them, and a significant fraction of toxins are structurally organized and combined with defined domains. Many toxin families have evolved by a process of gene duplication, with non-toxin paralogs typically being coexpressed in non-venom-gland tissues at low levels.
- Identification of Secretory Machinery: While the transcriptome identifies the toxin mRNA, it also reveals high expression of genes related to protein folding (e.g., protein disulfide isomerases) and processing, which are then detected as functional enzymes (e.g., chaperones) in the proteome.
- Venom plasticity: Venom composition is not static; it can shift during development (e.g., between newborn and adult snakes) or based on diet, suggesting a highly dynamic evolutionary response to environmental pressure.

These advances in general knowledge were only possible thanks to the integration of proteomic and transcriptomic approaches [10,11,12,13,14,15]. Genomic data is also important and complements the understanding of toxin evolutions [16,17].

However, major distinctions have also been observed:

- Discrepancy in Abundance: The transcriptome often shows high diversity with many rare transcripts, whereas the proteome is focused on the most active, highly expressed products.
- Post-Translational Modifications (PTMs): The proteome reveals modifications like C-terminal amidation and protein glycosylation that are predicted by the transcriptome but only confirmed as functional products in the venom itself.
- Isoform Variation: Transcriptomics may predict numerous protein isoforms (variants) due to alternative splicing, but only a fraction may be translated and detected in the venom proteome.

Altogether, these approaches are complementary in order to provide a more complete understanding of the structure, function and evolution of toxins in venom composition [14,15,16,17,18,19,20,21].

The knowledge provided by these studies may potentially lead to drug discovery and new therapeutics besides the major ones already in the market for:

- **Pain Management:** Identification of conotoxins from cone snails has led to drugs like Ziconotide.
- **Cardiovascular and Metabolic Disorder Treatments:** Identification of bradykinin and bradykinin-potentiating peptides in snake venom contributed to the development of ACE inhibitors (e.g., Captopril) and the study of new metabolic disorder (and aesthetic) treatments like Ozempic.

Rather than identifying single toxins, these omics studies offer a complete “snapshot” of the molecular arsenal of venomous organisms. In light of this, the published articles and reviews in this Special Issue, “**Transcriptomic and Proteomic Studies on Animal Venom: Looking Forward”,** provide new data and discussions generated by these technical approaches, contributing new findings on and extending the current knowledge to more venomous species, the complexity of venom mixtures, clinical, biochemical and toxic aspects, cellular responses induced by specific toxins, structural characterization of low-abundance venom transcripts, the role of extracellular vesicles in envenomation and pathophysiology, potential new achievements, and many other aspects. We hope that this Special Issue will contribute by shedding some light on the bright future of this important field of science.
